# Comparative Studies for Cryopreservation of *Agave* Shoot Tips by Droplet-Vitrification

**DOI:** 10.3390/plants13182609

**Published:** 2024-09-18

**Authors:** Lourdes Delgado-Aceves, Santiago Corona, Ubaldo Richard Marin-Castro, Martha Paola Rascón-Díaz, Liberato Portillo, Antonia Gutiérrez-Mora, María Teresa González-Arnao

**Affiliations:** 1 Centro de Investigación y Asistencia en Tecnología y Diseño del Estado de Jalisco, Unidad de Biotecnología Vegetal, Zapopan 45019, Jalisco, Mexico; madelgado_pos@ciatej.edu.mx; 2 Departamento de Botánica y Zoología, Centro Universitario de Ciencias Biológicas y Agropecuarias, Universidad de Guadalajara, Zapopan 44600, Jalisco, Mexico; pencasyquiotes@gmail.com (S.C.); liberato.portillo@cucba.udg.mx (L.P.); 3 Facultad de Ciencias Químicas, Universidad Veracruzana, Xalapa 91000, Veracruz, Mexico; umarin@uv.mx; 4 Centro de Investigación y Desarrollo en Alimentos, Xalapa 91190, Veracruz, Mexico; mrascon@uv.mx; 5 Facultad de Ciencias Químicas, Universidad Veracruzana, Orizaba 94340, Veracruz, Mexico

**Keywords:** survival, Differential scanning calorimetry, glass transition, histology, morphology, apical meristem

## Abstract

The objective of this work was to assess the suitability of the Droplet-vitrification protocol previously developed with *Agave peacockii* shoot tips for the cryopreservation of six *Agave* species. Shoot tips were precultured for 1 day on a medium with 0.3 M sucrose in the dark, loaded in a solution with 1.6 M glycerol and 0.4 M sucrose for 20 min, and dehydrated by exposure to Plant Vitrification Solution 2 (PVS2) at 0 °C for 20 min. Complementary studies using histological analysis, Differential scanning calorimetry (DSC), and evaluation of morphological characteristics in cryo-derived plants were performed. Survival rates ranged from 84% to 100% and from 76% to 97% before and after cryopreservation regardless of the *Agave* species belonging to two taxonomic subgenera. Thermal analysis of shoot tips subjected to the successive steps of the Droplet-vitrification protocol identified ice crystal formation after loading treatment and glass transition after osmotic dehydration with PVS2. The average glass transition temperature (Tg) was −55.44 °C based on the results of four *Agave* species. The histological studies showed the anatomical differences that could be found in the meristematic structures depending on the loss of apical dominance. This is the most advanced research on cryopreservation of *Agave* shoot tips.

## 1. Introduction

Many species of the *Agave* genus are used as raw materials to produce both distilled (“tequila”) and fermented beverages (“pulque,” “aguamiel”), as well as for the preparation of other types of syrup [1]. In addition, they are of ornamental interest, useful for the manufacture of agricultural substrates, for the textile industry and to produce paper by using the fibers of their leaves [2]. Consequently, *Agave* species represent an important source of income for the economy of different families and agricultural communities [3]. However, all these activities have caused the progressive degradation, displacement, and/or extinction of numerous agave populations [4,5,6]. Therefore, these species demand special attention to guarantee the safe conservation of their germplasm.

Cryopreservation is an important ex situ strategy for the long-term preservation of plant genetic resources [7,8]. Currently, this is the only technique available to ensure the safe and cost-effective conservation of different types of germplasm. Cryopreservation is based on the storage of biological material at ultra-low temperatures, usually that of liquid nitrogen (−196 °C) [9,10]. Various approaches have been successfully adapted to cryopreserve organized tissues, such as somatic embryos and shoot tips of different tropical and subtropical plant species [11,12]. In the case of the agave, somatic embryos of *Agave tequilana* F.A.C. Weber “Chato” [13] and shoot tips of *A. peacockii* were successfully cryopreserved using V-cryoplate technique [6] and droplet-vitrification method [14], respectively. However, any established protocol must be sufficiently operational for other species before its large-scale application.

According to Gentry [15], the genus *Agave* is classified into two subgenera: *Littaea* and *Agave*. This classification is based on the type of inflorescence they develop during the adult stage. The difference between the subgenera lies in their structure and gives rise to unique characteristics both in the genetics and morphology of the species.

In this work, different *Agave* species were selected to study their behavior during cryopreservation of shoot tips. The species were selected based on two criteria: taxonomic subgenus to which they belong: subgenus *Littaea* (*A. peacockii*, *A. rzedowskiana*, *A. victoriae-reginae*, *A. guiengola*) or subgenus *Agave* (*A. karwinskii*, *A. maximiliana*, *A. lurida*) and considering their current classification on the International Union for Conservation of Nature (IUCN) Red List: Least concern (*A. rzedowskiana*, *A. victoriae-reginae*, *A. maximiliana*); vulnerable (*A. peacockii*, *A. karwinskii*); endangered (*A. guiengola*); and extinct in the wild (*A. lurida*) [16].

Differential scanning calorimetry (DSC) is an important tool to perform complementary studies on thermal events during a cryopreservation process. This technique allows to support and standardize key factors involved in the development of a reliable protocol [17]. Furthermore, histological observations and morphological evaluations provide additional valuable information on the effects on tissue structures and their recovery capacity, which is crucial to understanding the success of cryopreservation.

The objective of this work was to validate the application of the Droplet-vitrification protocol previously developed with shoot tips of *A. peacockii* to cryopreserve shoot tips of six new *Agave* species of two taxonomic subgenera. Complementary studies were performed using Differential Scanning Calorimetry (DSC), histological analysis and morphological characterization to comparatively evaluate the post-cryopreservation response of shoot tips to the cryogenic procedure.

## 2. Materials and Methods

### 2.1. Plant Material

The biological material was provided by the Research Laboratory in Biotechnology and Restoration of Natural Environments from the University of Guadalajara. Shoots of *Agave peacockii*, *A. rzedowskiana*, *A. karwinskii*, *A. victoriae-reginae*, *A. maximiliana*, *A. lurida*, and *A. guiengola* species were introduced in vitro using Murashige and Skoog (MS) [18] semi-solid medium supplemented with L2 vitamins and 22.15 μM of 6-benzylaminopurine (BAP) for 60 days. Shoot elongation (4–5 cm in length) occurred for 90 days on a semi-solid MS medium devoid of regulators and was modified by reducing the NH_4_NO_3_ concentration to 5 mM [19].

All culture media contained 3% (*w/v*) sucrose and were solidified with 0.8% (*w/v*) agar (P-8169 Sigma^®^, St. Louis, MO, USA). The pH was adjusted to 5.8 ± 0.02, and the sterilization took place in an autoclave at 121 °C for 15 min and pressure of 1.3 kg cm^−2^. Cultures were incubated at 25 ± 2 °C and exposed to light intensity of about 27 µmol m^−2^ s^−1^ under a 16/8 h photoperiod (light/dark).

### 2.2. Cryopreservation of Agave Shoot Tips

Cryopreservation experiments were carried out using the Droplet-vitrification protocol described by Delgado-Aceves et al. [14] for *A. peacockii* shoot tips. Shoot tips (1 mm^3^) were aseptically removed from 4–5 cm long in vitro *Agave* plants using a Leica^®^ EZ4 HD stereoscope (Leica, Weitzler, Germany). After dissection, shoot tips were precultured in semi-solid MS medium supplemented with 0.3 M sucrose for one day, followed by exposure to a loading solution composed of basal MS medium with 0.4 M sucrose and 1.6 M glycerol for 20 min. Loaded tissues were osmotically dehydrated at 0 °C for 20 min exposed to Plant Vitrification Solution 2 (PVS2), comprising 30% (*w/v*) glycerol, 15% (*w/v*) ethylene glycol, 15% (*w/v*) dimethyl sulfoxide (DMSO), and 0.4 M sucrose [20]. For cooling, shoot tips were transferred to fresh drops of PVS2 placed on sterile aluminum foil strips, which were then introduced into 2 mL cryovials prefilled with liquid nitrogen (LN). Samples were kept in LN for 30 min. For rewarming, the aluminum strips were removed from the cryovials and quickly immersed into the unloading solution composed of MS basal medium supplemented with 1.2 M sucrose for 20 min. All experimental steps were performed under dark conditions.

After cryopreservation, explants were transferred to Petri dishes (5 cm diameter) containing basal MS semi-solid medium with 0.3 M sucrose in dark conditions. After 6 days, the explants were placed on basal MS medium supplemented with 500 mg L^−1^ glutamine, 250 mg L^−1^ casein hydrolysate, 3% (*w/v*) sucrose, and solidified with 0.8% (*w/v*) (recovery medium). Subsequently, the explants were transferred to a semi-solid-modified MS medium with a reduced concentration of NH_4_NO_3_ to 5 mM. Plant growth recovery took place under the same culture conditions previously described.

Ten shoot tips were considered per replicate, and three replicates were performed for treatment. Non-cryopreserved controls (−LN) were plants derived from elongation culture non-cryopreserved shoot tips, while cryopreserved controls (+LN) were those plants derived from shoot tips subjected to cryopreservation following the droplet vitrification protocol.

Survival was assessed as the percentage of shoot tips that showed elongation of the meristematic dome and displayed a green/yellow color 15 days after being recovered from LN exposure. Regrowth was expressed as the number of shoots that remained green and developed new leaves and shoots.

### 2.3. Shoot Histological Studies

The morphological structure of the shoot tips to be cryopreserved varies depending on the physiological stage in which tissues are at the time of dissection. To dissect meristems with similar anatomical characteristics, histological studies were performed before cryopreservation.

Tissue sections of shoots from *A. maximiliana* and *A. karwinskii* species isolated during the proliferation stage were used for histological observations. Tissue samples (around 0.5 × 0.5 cm^3^) were fixed using 70% *v/v* alcohol and embedded in polyethylene glycol (PEG, 1450 molecular mass) in a 1:4 proportion (PEG: deionized water) according to the protocol described by Burger and Richer [21]. Serial histological sections (15–18 µm) were obtained with a Leitz model 2155 rotary microtome. The slides were observed with a light microscope.

### 2.4. Differential Scanning Calorimetry

Differential Scanning Calorimetry (DSC) was used to characterize the thermal behavior of different *Agave* species at low temperatures and to study the effects of the successive steps of the Droplet-vitrification protocol [14] on phase transitions.

Fresh shoot tips of *Agave peacockii*, *A. rzedowskiana*, *A. karwinskii*, *A. victoria-reginae*, *A. maximiliana*, *A. lurida*, and *A. guiengola* were hermetically sealed in aluminum pans to prevent water evaporation before and during the evaluation cycles by DSC. The sample size ranged between 5 and 7 mg, and two repetitions per species were considered. In the same way, shoot tips of *A. peacockii*, *A. victoria-reginae*, *A. rzedowskiana*, *A. karwinskii*, and *A. maximiliana* were subjected to pre-growth and loading treatment, and then, compared with shoot tips pre-grown, loaded and exposed to PVS2.

Thermal analyses were performed using a DSC Q2000 V 23.4 equipment with a cooling system (RCS, −90 °C) and a DSC Discovery New series apparatus, both from TA Instruments, Inc., New Castle, DE, USA. The program included a cooling ramp of 10 °C min^−1^ from 35 to −70 °C and the same rate for heating from −70 °C to +35 °C. Melting temperature (Tm), enthalpy change (ΔH), and glass transition temperature (Tg) were processed using Universal Analysis Ver 3.9 software in DSC Q2000 and TRIOS V4 software in DSC Discovery, respectively. When the modulated mode was used, the modulation temperature amplitude was 1 °C over a period of 60 s. Moisture content (MC) was calculated by dividing the enthalpy change during fusion (ΔHm) by the fusion enthalpy of pure water (ΔHfus = 333.55 J/g).

### 2.5. Morphology Characterization of Agave In Vitro Plants

After six months (180 days) of in vitro culture, cryo-derived plants and in vitro-grown plants (non-cryopreserved controls) were evaluated for shoot length (cm), leaf shape, width-to-length ratio of leaves, and root length (cm). Morphological analysis was performed using five plants with three replicates for each study case: non-cryopreserved (−LN) and cryo-derived (+LN) plants.

### 2.6. Acclimatization

For acclimatization, obtained plants after six months (180 days) of in vitro culture were carefully transferred into a 50-hole seedling box containing peat moss and Perlite substrate (60:40 proportion). The plants were grown in a greenhouse at a temperature of between 14 and 35 °C, with a relative humidity of 30 to 40%, and exposed to natural light. The plants were watered twice a week as a practical recommendation.

### 2.7. Statistical Analyses

Cryopreservation experiments were replicated three times using 10 shoot tips per replicate. The dependent variable was survival before (−LN) and after (+LN) the immersion in LN. The results of cryopreservation assays were processed by one-way ANOVA, and before performing the ANOVA, the normality of the data distributions was verified. Means were compared by the least significant difference (LSD) range test with an error rate of *p* ≤ 0.05. All statistical analyses were carried out using the Minitab^®^ statistical software 17.2.1 (Minitab, State College, PA, USA).

**Figure 1:**
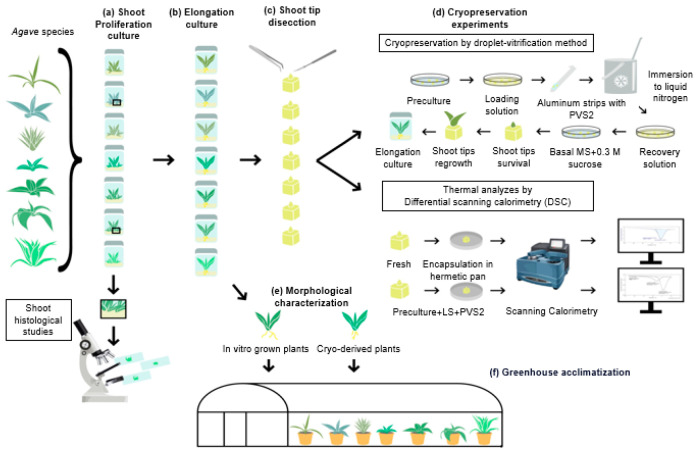
Experimental steps for the cryopreservation studies with *Agave*. (**a**) In vitro culture of *Agave* plants for 60 days on shoot proliferation medium and histological studies of tissues. (**b**) Elongation culture of the multiplied shoots to induce growth up to 4–5 cm in length. (**c**) Shoot tip (1 mm^3^) dissection. (**d**) Cryopreservation experiments and differential calorimetric analysis of *Agave* shoot tips. (**e**) Evaluation of morphological characteristics of in vitro grown (non-cryopreserved controls) and cryo-derived plants after 180 days of culture. (**f**) Greenhouse acclimatization for 12 months of plants derived from in vitro culture and from cryopreserved shoot tips.

The schematic representation of the cryobiological experiments developed with different *Agave* species is presented in Figure 1.

## 3. Results and Discussion

### 3.1. Cryopreservation of Agave Species Shoot Tips

**Figure 2:**
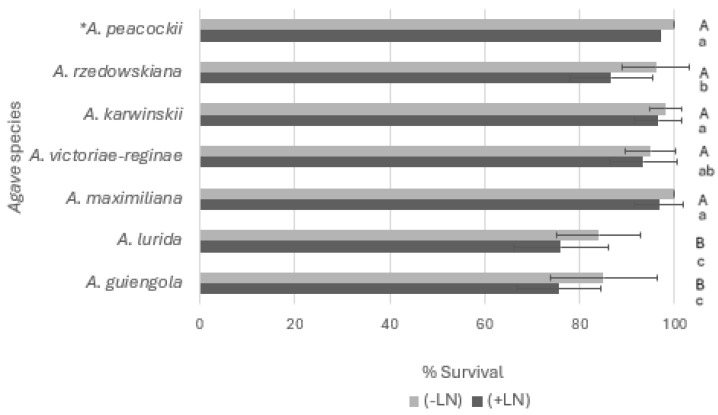
Effect of Droplet-vitrification protocol on survival (%) before (−LN) and after (+LN) cryopreservation of *Agave* shoot tips from different species. Results are presented as means ± SE, and different letters indicate significant differences (*p* < 0.05) analyzed by LSD multiple range test. Lowercase letters refer to non-cryopreserved controls (−LN), and uppercase letters refer to cryopreserved (+LN) samples. * Data reference (Delgado-Aceves et al., 2022) [14].

The results of our experiments demonstrated the successful application of the Droplet-vitrification protocol for the cryopreservation of shoot tips from different *Agave* species (Figure 2). Shoot tip survival remained sufficiently high regardless of the *Agave* species used and ranged from 84% to 100% (on average 94%) before immersion in LN and from 76% to 97% (on average 89%) after cryopreservation. Overall, these findings also indicated the high tolerance of species of this genus. The significantly lower responses always exceeded 75% survival with shoot tips of only two (*A. lurida* and *A. guiengola*) of the seven evaluated species. These results, therefore, provide a new biotechnological alternative useful for safeguarding the germplasm of exceptional species classified on the Red List as extinct in the wild and endangered, respectively.

It is important to note that the standard deviations associated with the survival rates show variability which can be attributed to the inherent biological variation between individual plants, as well as to species-specific factors such as genotype. However, the subgenus to which the species belong apparently does not influence the response to the cryogenic process because the survival rates of *Agave guiengola* (subgenus *Littaea*) and *Agave lurida* (subgenus *Agave*) were the same and less than 80% regardless of the subgenus. Thus, the differential characteristics between both subgenera only impact the morphology of their floral structure: *Littaea* presents spiked flowers in pairs or sometimes in small clusters, meaning that the flowers are directly attached to the inflorescence. In contrast, *Agave* has paniculate flowers in large umbellate clusters on lateral peduncles, showing the inflorescence with lateral branches from which the flowers emerge [15].

On the other hand, one could also hypothesize that some ecological factors could be related to the behavior after cryopreservation. However, even though the two species are endemic to the same region, they both grow under different conditions: *A. guiengola* grows between 100 and 1000 m in limestone formations, and *A. lurida* grows in volcanic rocky soils in semi-arid tropical forests at an altitude of approximately 1850 m (Gentry, 1982) [15]; therefore, differences in ecological growth conditions apparently did not significantly influence either.

Another interesting factor that could be conditioning the response to cryopreservation could be related to mucilage cells. Zhang et al. [22] reported a close relationship between mucilage cells and tolerance to low temperatures in succulent plants of *Sedum aizoon* L. Therefore, it would be useful to study other characteristics in the *Agave* species (i.e., fiber density, mucilage, metabolite content) in future research.

**Figure 3:**
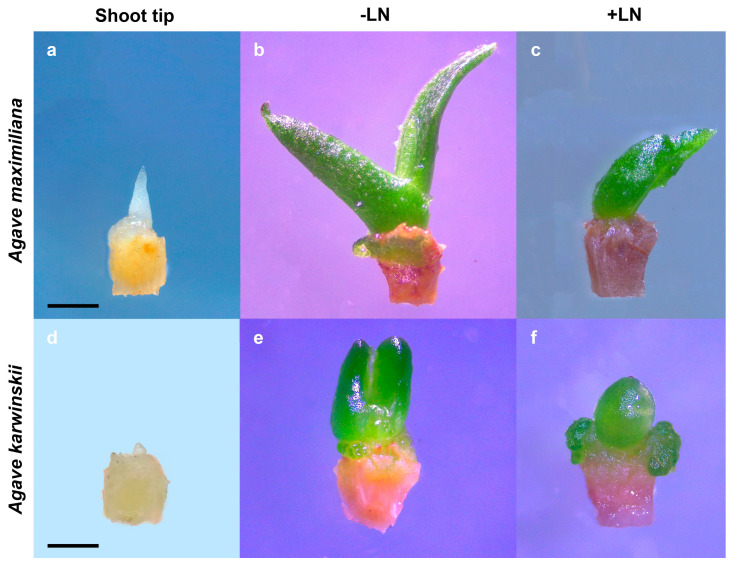
Regrowth of *Agave* shoot tips after 30 days of culture. (**a**,**d**) Fresh shoot tips of *Agave maximiliana and A. karwinskii*; (**b**,**e**) (−LN) regrowth of shoot tips precultured in 0.3 M sucrose for 1 day in the dark and loaded with solution (0.4 M sucrose and 1.6 M glycerol for 20 min) and osmotically dehydrated with Plant Vitrification Solution 2 (30% (*w/v*) glycerol, 15% (*w/v*) ethylene glycol, 15% (*w/v*) DMSO (dimethyl sulphoxide) and 0.4 M sucrose); (**c**,**f**) (+LN) regrowth of pretreated and cryopreserved shoot tips. Bar = 1 mm.

Our study also revealed notable disparities in the initial growth rates between non-cryopreserved and cryopreserved shoot tips. The cryopreserved apical meristems exhibited a slightly slower growth compared to the controls (Figure 3), suggesting the effect of cryopreservation on regenerative capacity and delay in the development trajectory of plant tissues even when the optimized protocol is applied [23].

**Figure 4:**
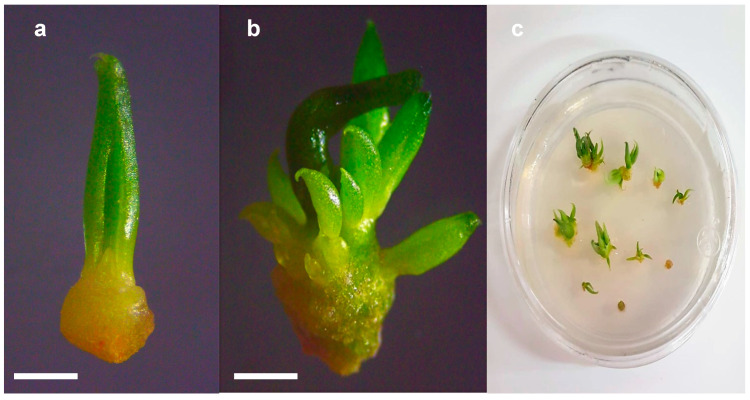
Regrowth of cryopreserved *Agave peacockii* shoot tip. (**a**) Regrowth of cryopreserved shoot tip after 30 d of culture. (**b**) Morphogenic event of multiple shoot formation. (**c**) Shoot proliferation 60 d after cryopreservation. Bars = 1 mm.

Once the first delay in growth is overcome, the following detected morphogenetic process corresponds to the formation of multiple buds. This event is characterized by the production of adjacent tissues to the apical meristem (Figure 4). Principal bud dominance can be observed (brown-green tissue) as well as new buds (green-young tissue) formation around it. The endogenous accumulation of growth regulator during the micropropagation of donor plants is also expressed in the explant by the proliferation of axillary shoots (Figure 4b,c). These results also indicate the importance of observing and adjusting the micropropagation method, especially because less genetic variation is desired since each species generates it differently [24,25,26].

### 3.2. Shoot Histological Studies

**Figure 5:**
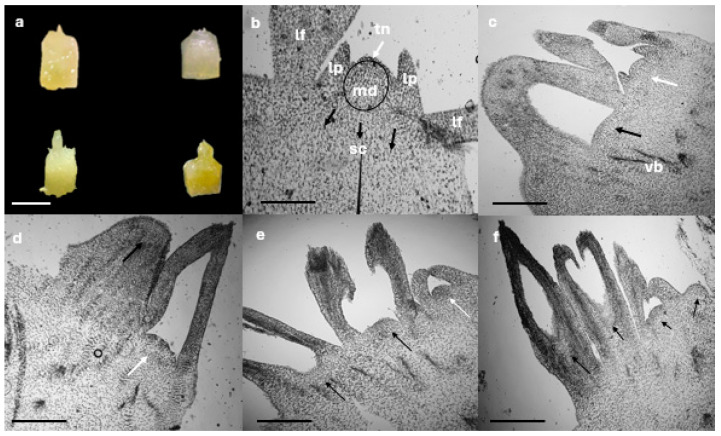
In vitro formation of *Agave* shoot tips. (**a**) Shoot tips with different morphology excised from in vitro grown plants for 90 days. (**b**) Shoot tips of *A. maximiliana* showing optimal morphology for cryopreservation; tunica (white arrow) and secondary shoot tip cells (black arrows indicate large intracellular spaces). (**c**) Shoot tip of *A. maximiliana* with a well-formed meristematic dome (white arrow) and the tip without a meristematic dome region (black arrow). (**d**) Apical meristem of *A. maximiliana* without an organized meristematic dome and presenting mature leaves (white arrow) and elongation of dome meristematic (black arrow). (**e**) Apical meristem of *A. karwinskii* showing optimal morphology (white arrow) and apical meristems with the absence of an organized meristematic dome with elongated leaf primordia (black arrow). (**f**) Shoot tips in different stages of development. md = meristematic dome, sc = secondary cells, lp = leaf primordia, lf = lateral young leaf, tn = tunica, vb = vascular bundles Bars: a = 1 mm, b–f = 200 µm.

It has been observed that the quality, development, and size of the initial meristem are important factors in the success of cryopreservation [27]. In the case of the apical meristems of *A. maximiliana* and *A. karwinskii*, differences in morphology and development were detected, even though in vitro plants had been subjected to the same culture conditions. Histological studies allowed us to observe the anatomical differences and the possible stages in which the meristematic structures can be found before the shoot tip dissection (Figure 5). Several lateral buds did not present a good definition of the meristematic tissues. The presence of lateral buds suggests the loss of apical dominance, which may be affecting the development of the shoot tip [28]. (Figure 5c,d,f).

For cryopreservation, the best morphological features of shoot tips are described as a meristematic dome with leaf primordia measuring 1 mm long × 1 mm wide [14]. Small explant sizes facilitate the absorption of the cryoprotectants and, consequently, improve water removal [27]. Therefore, a fundamental biological factor is the good formation of the apical meristem, which is characterized by a meristematic dome with isodiametric cells, dense cytoplasm, small vacuole, defined nuclei, few vascular bundles, and two primordial leaves in good condition (Figure 1b) [14,27,28,29].

Therefore, shoot tips without a well-defined meristematic dome region can be considered unsuitable for extraction.

Morphological studies by Souza et al. [30] and Guerra et al. [28] showed that cryo-injuries mainly occurred in cells that were already differentiated and had low density. These authors reported that pineapple shoot tips with these characteristics did not survive cryopreservation.

### 3.3. Differential Scanning Calorimetry

Thermal analysis showed that the initial moisture content of fresh shoot tips was mostly higher than 70%, with great similarity among *Agave* species. (Table 1).

**Figure 6:**
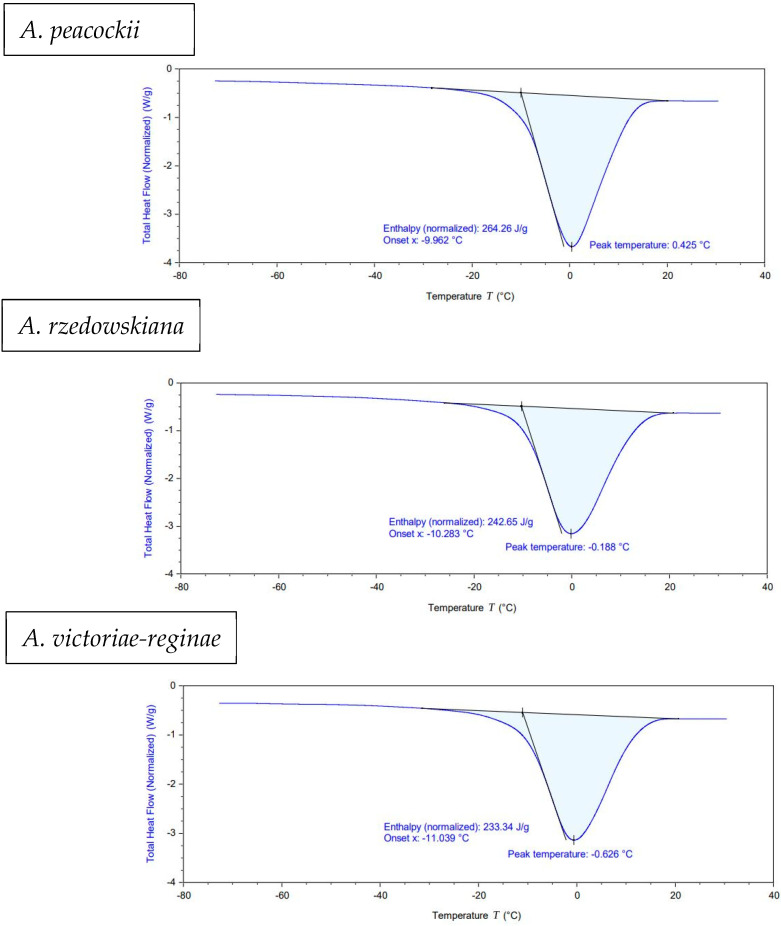
Representative thermograms in the heating phase during the analysis of *A. peacockii*, *A. rzedwiskiana*, and *A. victoriae-reginae* fresh shoot tips.

Examples of thermograms with fresh shoot tips of *A. peacockii*, *A*. *rzedwiskiana*, and *A. victoriae-reginae* species are shown in Figure 6.

When the shoot tips were subjected to the successive steps of the Droplet-vitrification protocol, heating thermograms showed that after the loading treatment, melting temperatures (*T_m_*) and enthalpies (Δ*H*) decreased due to the reduction (about 50%) of the initial MC. At this stage, the thermal first-order event was always identified by the endothermic peak of melting. However, following PVS2 exposure, the glass transition became evident, and fusion transitions were no longer observed regardless of the *Agave* species (Table 2). The calculated average using the identified Tg values in modulated mode was −55.44 °C. This average is based on analyses performed with shoot tips of *A. peacockii* (Tg: −58.02 °C), *A. victoriae-reginae* (Tg: −53.56 °C), *A. rzedowskiana* (Tg: −54.29 °C), and *A. karwinskii* (Tg: −55.99 °C).

**Figure 7:**
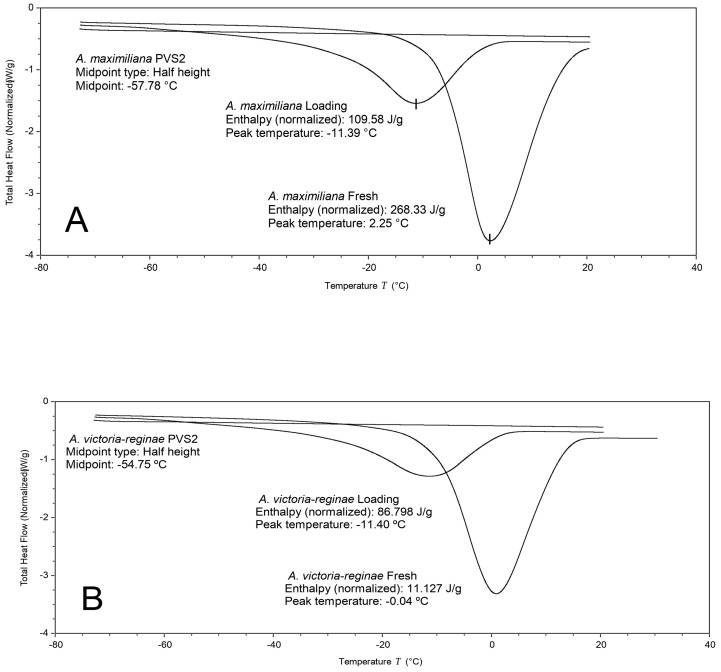
DSC analysis of *Agave* shoot tips subjected to the different steps of the Droplet-vitrification protocol. (**A**) *A. maximiliana*, (**B**) *A. victoriae-reginae*.

Thermal analysis of the different shoot tip samples allowed us to distinguish two types of phase transitions at low temperatures: ice crystal formation and vitrification. DSC assessments also showed how the osmotic dehydration by exposure to PVS2 had a decisive effect on glass transition induction (Figure 7). Vitrification is a key physical process to prevent lethal intracellular ice formation that produces irreversible lesions in membranes, cells, and organelles [31]. This event is essential to successfully cryopreserve plant germplasm [32]. According to our experiments, the occurrence of vitrification after exposure to PVS2 may explain why survival rates (84–100% survival) remained high after immersion in liquid nitrogen (76–97% survival).

DSC analysis represents a powerful tool to support fundamental studies that help the development and/or optimization of cryopreservation protocols. This technique also contributes to the better interpretation of experimentally obtained results [11,17,33]. Based on thermodynamic principles, we were able to understand, from another analytical perspective, the successful application of the previously developed protocol [14] to a larger number of *Agave* species.

### 3.4. Morphological Characterization of Agave In Vitro Plants

The comparison of morphological characteristics between non-cryopreserved controls and cryo-derived plants under in vitro culture conditions is presented in Table 3.

Non-cryopreserved controls produced significantly larger shoots than those regenerated from cryopreserved samples, except for the species *A. peacockii* [14], for which no differences were found with respect to this morphological indicator. However, despite the initial delay in growth, shoot tips recovered after cryopreservation grew and developed normal plantlets and roots, regardless of the *Agave* species used.

It is well known that plants subjected to cryopreservation may experience changes in their growth and development compared to control plants [7]. The differences in growth rate are usually manifested in various morphological aspects such as plant height, size of leaves, branching patterns, and overall vigor. Cryopreserved plants may exhibit stunted growth, smaller leaf size, and reduced branching compared to controls. Additionally, variations in root development and architecture may also occur in cryopreserved plants [23].

These morphological differences between controls and cryopreserved samples can be attributed to the stress induced by the cryopreservation process, first due to treatments with cryoprotective agents and then to cooling in liquid nitrogen [34]. The physiological changes that occur during cryopreservation, such as cellular damage and altered hormone levels, can affect subsequent growth and development. Understanding the extent of morphological variation between control and cryopreserved plants is crucial for assessing the overall impact of cryopreservation on plant physiology and morphology [23].

On the other hand, in vitro culture can significantly accelerate the growth of some species compared to their growth in the wild. For instance, *A. rzedowskiana* is known for its slow growth, reaching only 40 cm in height in adulthood after more than 30 years [35]. However, in vitro, this species exhibits notably accelerated growth and shows an ability to develop axillary shoots even without the addition of growth regulators [14].

Another notable case is *A. victoriae-reginae*, which in the wild takes approximately 20–30 years to reach full vegetative growth and rarely produces more than 2–3 rhizomatous shoots throughout its lifespan. In contrast, the in vitro culture of this species demonstrates highly proliferative behavior without the need for growth regulators [36].

In this sense, cryo-derived plants acclimated in the greenhouse return the behavior to that of a wild species without the production of axillary buds and slow growth. Leaf shape, rosette structure, and presence of terminal spine were observed, and no apparent change was observed that would show any anomaly in its development.

**Figure 8:**
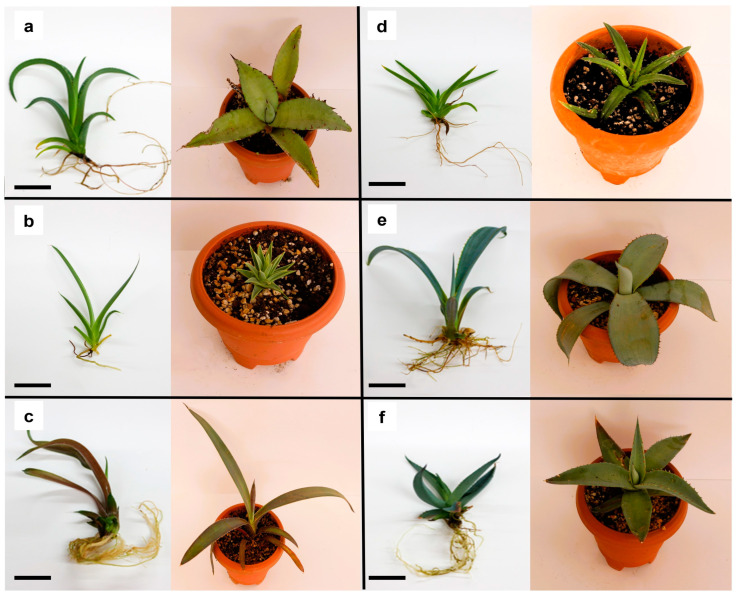
Morphological comparison between agave vitroplants (**left**) and acclimatized plants (**right**) of 12 months of age under greenhouse cultivation conditions: (**a**) *A. peacockii*, (**b**) *A. rzedowskiana*, (**c**) *A. karwinskii*, (**d**) *A. victoriae-reginae*, (**e**) *A. maximiliana*, and (**f**) *A. guiengola.* Bars = 1 cm.

To detect any abnormality in the development and growth of each species, it is necessary to know the dimensions that plants can reach in the natural environment. This could be an indicator of the time needed to reach the size characteristic of the species. The shape of the leaf and the structure of the rosette are also important in the case of agaves. In vitro *Agave* plants may display abnormal morphology, such as elongated stems or leaves, and reduced pigmentation due to the absence of environmental cues. Acclimated agave plants generally exhibit a more natural morphology, including compact rosette formation, well-developed leaves, and adequate pigmentation in response to light exposure (Figure 8).

In summary, the studies developed on cryopreservation of *Agave* shoot tips confirmed the usefulness of the Droplet-vitrification protocol, which has also been reported for many other species of different tropical crops such as potato, ornamentals [37], garlic [38], and pineapple [28].

## 4. Conclusions

This work provided new technological information and applicable results for the extension of cryopreservation to different *Agave* species. Our findings confirmed that the Droplet-vitrification method is an efficient procedure for the long-term conservation of *Agave* shoot tips for safeguarding the germplasm of exceptional species classified on the Red List.

The previously defined protocol with *A. peacockii* shoot tip was successfully applied to six additional *Agave* species of two taxonomic subgenera, demonstrating that it could be implemented as a reproducible and routine technique in gene banks, botanical gardens, and scientific institutes that have created cryobanks for their research.

Histological analysis revealed all possible meristematic structures that could be found before shoot tip dissection. These observations allowed us to identify the best anatomical and morphological features that shoot tips should have to be successfully cryopreserved regardless of the species or genotype. In this sense, in vitro donor plants must be previously cultured in the absence of growth regulators to induce greater plant elongations and favor a better definition of the meristematic region. The presence of lateral buds influences the loss of apical dominance and interferes with the good development of the shoot tips.

This is the first report on the thermal behavior of shoot tips of the *Agave* genus. DSC studies allowed us to identify the water phase transitions in the shoot tips by the effect of the successive steps of the Droplet-vitrification protocol. The interpretation of the thermal profiles demonstrated the significant effect of dehydration with PVS2 on the induction of glass transition at an average Tg of −55.44 °C. These results evidenced ice melting events after loading treatment for 20 min despite the reduction of the initial moisture content by around 50% and suggested that the same exposure time to PVS2 was apparently sufficient to completely remove free water and promote vitrification events.

Despite the slower growth of cryopreserved agave plants compared to non-cryopreserved in vitro controls, no anomalies were observed in the morphological characteristics evaluated in vitro or during the subsequent development of plants under greenhouse conditions.

In conclusion, this work contributed to generating greater practical knowledge on the cryopreservation of *Agave* shoot tips and constitutes a new step forward for its future large-scale application.

## Figures and Tables

**Table 1:** DSC Analysis of fresh shoot tips from different *Agave* species.

Species	$T_{m}$ * (°C)	$\Delta H_{m}$ ** (J/g)	Moisture Content (%)
*A. peacockii*	−0.332 ± 1.07	251.23 ± 18.42	75.32 ± 5.52
*A. rzedwiskiana*	0.423 ± 0.86	238.72 ± 5.55	71.57 ± 1.66
*A. karwinskii*	0.131 ± 0.65	244.52 ± 2.99	73.08 ± 0.89
*A. victoriae-reginae*	−0.408 ± 0.30	226.42 ± 9.78	67.88 ± 2.93
*A. maximiliana*	1.885 ± 1.04	267.32 ± 1.42	80.14 ± 0.42
*A. lurida*	0.236 ± 0.24	255.60 ± 6.56	76.63 ± 1.96
*A. guiengola*	0.500 ± 0.22	254.23 ± 5.13	76.21 ± 1.53

* Melting temperature; ** change of enthalpy; fusion Enthalpy of pure water (ΔHfus = 333.55 J/g).

**Table 2:** DSC analysis of *Agave* shoot tips subjected to different steps of the Droplet-vitrification protocol.

Species	Treatment	$T_{m}\;$* (°C)	$\Delta H_{m}$ ** (J/g)	Moisture Content (%)
*A. peacockii*	Fresh	−0.332 ± 1.07	251.23 ± 18.42	75.32 ± 5.52
	Pregrowth + Loading	−10.64 ± 0.73	117.86 ± 20.73	35.33 ± 6.21
	Pregrowth + Loading + PVS2	-	ND	-
*A. rzedwiskiana*	Fresh	0.423 ± 0.86	238.72 ± 5.55	71.57 ± 1.66
	Pregrowth + Loading	−6.16 ± 0.50	122.50 ± 12.02	36.72 ± 3.60
	Pregrowth + Loading + PVS2	-	ND	-
*A. karwinskii*	Fresh	0.131 ± 0.65	244.52 ± 2.99	73.08 ± 0.89
	Pregrowth + Loading	−8.10 ± 1.23	146.30 ± 28.14	43.86 ± 8.43
	Pregrowth + Loading + PVS2	-	ND	-
*A. victoriae-reginae*	Fresh	0.100 ± 1.00	240.60 ± 10.27	72.12 ± 3.08
	Pregrowth + Loading	−10.25 ± 1.62	97.97 ± 15.79	29.37 ± 4.73
	Pregrowth + Loading + PVS2	-	ND	-

* Melting temperature; ** change of enthalpy. ND: No thermal first-order event detected.

**Table 3:** Comparison of morphological characteristics between non-cryopreserved control plants (−LN) and cryo-derived (+LN) plants after 180 days of in vitro culture.

*Agave* Species	Condition	Shoot Length (cm) **	Leaf Shape	*W*/*V* *	Root Length (cm) **
*A. peacockii*	−LN	5.97 ± 0.90 a	Linear-lanceolate	2.66 ± 0.66 a	16.19 ± 2.58 a
	+LN	5.28 ± 1.11 a		1.83 ± 0.73 b	10.33 ± 1.64 b
*A. rzedowskiana*	−LN	6.08 ± 1.01 a	Linear-triangular	0.23 ± 0.05 b	7.00 ± 2.37 a
	+LN	4.78 ± 0.67 b		0.82 ± 0.25 a	3.53 ± 1.94 b
*A. karwinskii*	−LN	9.15 ± 2.02 a	Linear-lanceolate	5.27 ± 1.57 a	6.06 ± 2.53 a
	+LN	5.94 ± 1.09 b		2.77 ± 1.08 b	3.91 ± 1.69 b
*A. victoriae-reginae*	−LN	4.79 ± 0.90 a		1.25 ± 0.41 a	14.22 ± 5.37 a
	+LN	2.15 ± 0.98 b	Lineal-ovate	0.34 ± 0.21 b	3.78 ± 1.60 b
*A. maximiliana*	−LN	9.22 ± 2.26 a	Lanceolate or oblacnceolate	11.45 ± 5.48 a	4.25 ± 1.87 a
	+LN	5.46 ± 1.11 b		3.43 ± 1.27 b	3.10 ± 0.73 b
*A. guiengola*	−LN	4.38 ± 0.75 a	Ovate-ovate lanceolate	1.97 ± 0.51 a	9.47 ± 2.44 a
	+LN	1.17 ± 0.60 b		0.13 ± 0.97 b	-

* *W/V* represents the width/length of leaves, five plants with three replicates per test. ** Results are presented as means ± SE, and different letters within the columns are significantly different according to the LSD test (*p* ≤ 0.05). (–) No roots.

## Data Availability

The original contributions presented in the study are included in the article, further inquiries can be directed to the corresponding author.

## References

[1] García-Mendoza A., Nieto-Sotelo J., Sánchez-Teyer L., Tapia E., Gómez-Leyva J., Tamayo-Ordoñez M.C. (2017). Panorama del Aprovechamiento de los Agaves en Mexico. AGARED—Red Temática Mexicana Aprovechamiento Integral Sustentable y Biotechnología de los Agaves.

[2] Pérez-Zavala M.D.L., Hernández-Arzaba J.C., Bideshi D.K., Barboza-Corona J.E. (2020). Agave: A natural renewable resource with multiple applications. J. Sci. Food Agric..

[3] Alducin-Martínez C., Ruiz Mondragón K.Y., Jiménez-Barrón O., Aguirre-Planter E., Gasca-Pineda J., Eguiarte L.E., Medellin R.A. (2023). Uses, Knowledge and Extinction Risk Faced by *Agave* Species in Mexico. Plants.

[4] Colunga-GarcíaMarín P., Zizumbo-Villarreal D. (2007). Tequila and other *Agave* spirits from west-central Mexico: Current germplasm diversity, conservation and origin. Biodivers. Conserv..

[5] Torres I., Casas A., Vega E., Martínez-Ramos M., Delgado-Lemus A. (2015). Population dynamics and sustainable management of mescal Agaves in central Mexico: *Agave potatorum* in the Tehuacán Cuicatlán valley. Econ. Bot..

[6] Delgado-Aceves M.L. (2022). Desarrollo de Estrategias Para el Cultivo *In Vitro* y Crioconservación de Especies de *Agave* (*A. tequilana* cultivar ‘Chato’, *A. peacockii* y *A. cupreata*). Ph.D. Thesis.

[7] Wang M.-R., Bi W., Shukla M.R., Ren L., Hamborg Z., Blystad D.-R., Saxena P.K., Wang Q.-C. (2021). Epigenetic and Genetic Integrity, Metabolic Stability, and Field Performance of Cryopreserved Plants. Plants.

[8] Pence V.C., Bruns E.B. (2024). Scratching the surface: The *in vitro* research that will be critical for conserving exceptional plants to scale. In Vitro Cell. Dev. Biol.-Plant.

[9] González-Arnao M.T., Martínez-Montero M., Cruz-Cruz C.A., Engelmann F., Ahuja M.R., Ramawat K.G. (2014). Advances in cryogenic techniques for the long-term preservation of plant biodiversity. Biotechnology and Biodiversity.

[10] Benelli C. (2021). Plant Cryopreservation: A Look at the Present and the Future. Plants.

[11] González-Arnao M.T., Gámez R., Martínez Y., Valdés S., Mascorro J.O., Osorio A., Pastelín M., Guevara M., Cruz C.A., González-Arnao M.T., Engelmann F. (2013). Estado actual de la Crioconservación vegetal en México. Crioconservación de Plantas en América Latina y el Caribe.

[12] Kaviani B., Kulus D. (2022). Cryopreservation of Endangered Ornamental Plants and Fruit Crops from Tropical and Subtropical Regions. Biology.

[13] Delgado-Aceves L., González-Arnao M.T., Santacruz-Ruvalcaba F., Folgado R., Portillo L. (2021). Indirect Somatic Embryogenesis and Cryopreservation of *Agave tequilana* Weber Cultivar ‘Chato’. Plants.

[14] Delgado-Aceves M.L., Portillo L., Folgado R., Romo-Paz F.J., González-Arnao M.T. (2022). New approaches for micropropagation and cryopreservation of *Agave peacockii*, an endangered species. Plant Cell Tissue Organ Cult..

[15] Gentry H. (1982). Agaves of Continental North America.

[16] International Union for the Conservation of Nature (2024). The IUCN Red List of Threatened Species. Version 2024-1. https://www.iucnredlist.org.

[17] Zámečník J., Faltus M., Bilavčík A., Kotková R., Katkov I. (2012). Comparison of cryopreservation methods of vegetatively propagated crops based on thermal analysis. Current Frontiers in Cryopreservation.

[18] Murashige T., Skoog F. (1962). A revised medium for rapid growth and bioassays with tobacco tissue cultures. Physiol. Plant..

[19] Castro-Concha L., Loyola-Vargas V.M., Chan J.L., Robert M.L. (1990). Glutamate dehydrogenase activity in normal and vitrified plants of *Agave tequilana* Weber propagated in vitro. Plant Cell Tissue Organ Cult..

[20] Sakai A., Kobayashi S., Oiyama I. (1990). Cryopreservation of nucellar cells of navel orange (*Citrus sinensis* Osb. var. brasiliensis Tnaka) by vitrification. Plant Cell Rep..

[21] Burger L., Richter H. (1991). Anatomia da Madeira.

[22] Zhang R., Chen Y., Yang L., Xie Y., Yan W., He Z. (2020). Study on the relationship between mucilage cells and low temperature tolerance of *Sedum aizoon* L.. IOP Conf. Ser. Earth Environ. Sci..

[23] Popova E., Kulichenko I., Kim H.-H. (2023). Critical Role of Regrowth Conditions in Post-Cryopreservation of In Vitro Plant Germplasm. Biology.

[24] Hernández A., Palacios H., Portillo L., Gutiérrez-Mora A. (2014). Histologic origin of micropropagated shoots of *Agave tequilana*. Sustainable and Integral Exploitation of Agave.

[25] Delgado-Aceves L., Palacios H., Romo-Paz F., Portillo L., Gutiérrez-Mora A. (2019). Micropropagation systems in *Agave* spp.: Common errors. Integral and Sustainable Use of Agave.

[26] Bettoni J., Chen K., Volk G., Volk G., Balunek E., Chen K. (2020). An Overview of Shoot Tip Cryopreservation. Training in Plant Genetic Resources: Cryopreservation of Clonal Propagules.

[27] Bettoni J.C., Wang M.R., Li J.W., Fan X., Fazio G., Hurtado-Gonzales O.P., Volk G.M., Wang Q.C. (2024). Application of biotechniques for *in vitro* virus and viroid elimination in pome fruit crops. Phytopathology.

[28] Guerra P.A., Souza E.H., Max D.A.S., Rossi M.L., Villalobos-Olivera A., Ledo C.A.S., Martinez-Montero M.E., Souza F.V.D. (2021). Morphoanatomical aspects of the starting material for the improvement of pineapple cryopreservation by the droplet-vitrification technique. An. Acad. Bras. Cienc..

[29] Engelmann F. (2011). Use of biotechnologies for the conservation of plant biodiversity. In Vitro Cell. Dev. Biol. Plant.

[30] Souza F.V.D., Kaya E., Vieira L.J., Souza E.H., Amorim V.B.O., Skogerboe D., Matsumoto T., Alves A.A.C., Ledo C.A.S., Jenderek M.M. (2016). Droplet-vitrification and morphohistological studies of cryopreserved shoot tips of cultivated and wild pineapple genotypes. Plant Cell Tissue Organ Cult..

[31] Faltus M., Domkářová J., Svoboda P., Horáčková V., Nesvadba V., Klička V., Ptáček J., Bilavcik A., Zamecnik J. (2024). Analysis of Thermal Characteristics of Potato and Hop Pollen for Their Cryopreservation and Cross-Breeding. Plants.

[32] Sakai A., Engelmann F. (2007). Vitrification, encapsulation-vitrification and droplet-vitrification: A review. CryoLetters.

[33] Benson E.E., Reed B.M., Brennan R.M., Clacher K.A., Ross D.A. (1996). Use of Thermal Analysis in the Evaluation of Cryopreservation Protocols for Ribes nigrum L. Germplasm. CryoLetters.

[34] Smulders M.J.M., de Klerk G.J. (2011). Epigenetics in plant tissue culture. Plant Growth Regul..

[35] Carrillo-Reyes P., Aviña R.V., Ramírez-delgadillo R. (2003). *Agave rzedowskiana*, a new species in subgenus Littaea (Agavaceae) from western Mexico. Brittonia.

[36] Rodríguez-Garay B., Gutiérrez-Mora A., Acosta-Dueñas B. (1996). Somatic embryogenesis of *Agave victoria-reginae* Moore. Plant Cell Tissue Organ Cult..

[37] Normah M.N., Sulong N., Reed B.M. (2019). Cryopreservation of shoot tips of recalcitrant and tropical species: Advances and strategies. Cryobiology.

[38] Senula A., Nagel M. (2021). Cryopreservation of Plant Shoot Tips of Potato, Mint, Garlic, and Shallot Using Plant Vitrification Solution 3. Methods Mol. Biol..

